# Optimizing a modified cetyltrimethylammonium bromide protocol for fungal DNA extraction: Insights from multilocus gene amplification

**DOI:** 10.1515/biol-2022-1006

**Published:** 2025-02-03

**Authors:** Gulam Jeelani Dar, Ruqeya Nazir, Shakil A. Wani, Saleem Farooq, Tariq Aziz, Thamer H. Albekairi

**Affiliations:** Centre of Research for Development (CORD), University of Kashmir, Srinagar 190006, Jammu and Kashmir, India; Bacteriology Laboratory, Division of Veterinary Microbiology & Immunology, SK University of Agricultural Sciences and Technology of Kashmir, Srinagar, India; Department of Environmental Science, University of Kashmir, Srinagar 190006, Jammu and Kashmir, India; Laboratory of Animal Health, Food Hygiene and Quality, University of Ioannina, Ioannina, Greece; Department of Pharmacology and Toxicology, College of Pharmacy, King Saud University, Riyadh, Saudi Arabia

**Keywords:** CTAB, gene loci, genomic DNA, ITS, MLST, PCR

## Abstract

Genomic DNA (gDNA) extraction is an important step in many molecular studies of fungal biology, and it is necessary to evaluate the efficiency, cost-effectiveness, and efficacy of different extraction methods to ensure successful amplification of the target gene and minimize deoxyribonucleic acid (DNA) degradation. The modified cetyltrimethylammonium bromide (CTAB) method was found to be effective in releasing high molecular weight gDNA with minimal protein contamination. Based on anticipated gDNA yield and quality, extraction time, cost effectiveness, successful amplification, and waste management, our findings serve as a guide for selecting techniques of gDNA extraction from fungal species. This study presents a modified CTAB method for extracting DNA from a variety of fungal species including *Aspergillus*, *Penicillium*, *Alternaria*, *Dothiorella,* and *Fusarium*. Comparison of three cell crushing methods reveals similar gDNA yields, demonstrating the method’s effectiveness. Furthermore, the modified CTAB method is cost-effective and safe, eliminating the need for grinding with liquid nitrogen or bead beating. The method has a potential use for nucleic-based fungal disease diagnosis such as fish fungal diseases, plant pathogens, fruit rot associated pathogens, and human fungal diseases as we were successful in PCR amplifying several gene loci from varied fungal pathogens.

## 1 Introduction

Deoxyribonucleic acid (DNA) extraction is a crucial step in fungal molecular systematics [1], clinical diagnostics [2], phylogenetic analysis [3,4], evolutionary research [5,6], genetics, and genomics [7,8,9,10]. Despite the fact that all DNA extraction procedures differ in yield and quality, the integrity and quality of extracted DNA will influence the accuracy of sequencing results [11,12,13,14]. Correspondingly, any DNA extraction protocol is distinguished by the qualities of enhanced DNA yield, diminished DNA degradation, and high-quality DNA for subsequent DNA analysis procedures including polymerase chain reaction (PCR) amplification and Multilocus Sequence Typing (MLST). MLST is used to detect minute variations in the genomes of fungal species using multiple gene haplotypes [15]. So, DNA extraction protocol must have the above attributes for MLST analysis [16,17,18]. Moreover, besides downstream processing competence, the DNA extraction protocol should be inexpensive, expeditive, and produce a lesser amount of perilous waste [19,20,21].

Within the scientific literature, there has been a dearth of an effective DNA extraction methodology for the molecular characterization of fungal species utilizing multiple gene loci. The main challenge is to develop a technology that is both fast and sensitive enough to work with modest quantities of DNA from a small number of cells. Disrupting the cell wall without damaging the genomic DNA (gDNA) is one of the most challenging procedures in isolating fungal gDNA [22]. Cultures grown specifically in liquid media were utilized to extract DNA from fungal isolates. However, because the inhibitors in the agar might interfere with DNA extraction, cultures grown on agar are rarely employed for direct isolation [23].

HiPura^TM^ Fungal DNA Purification Kit [24] and DNA Easy (Qiagen kit) [DNeasy] Plant Mini Kit, Qiagen [25] were employed to assess the efficacy of extraction and quality of gDNA extracted from fungal species using modified cetyltrimethylammonium bromide (CTAB) protocol. Several modified CTAB DNA extraction protocols are currently in vogue in a variety of research fields, including fungal ecology [26], fungal phylogenetics [27,28], and forensic studies employing fungi [29]. The CTAB [30] method is time-consuming, expensive, and interferes with efficient PCR amplification of genetic markers. It involves gradient centrifugation of cesium chloride-ethidium bromide (EtBr) to extract high-quality DNA. Additional changes are needed because EtBr is a mutagenic agent with scant data supporting its use in fungal DNA extraction [31].

In the modified CTAB DNA extraction protocol, gDNA is initially freed by lysing the cells in the presence of a gDNA stabilizing agent with an anionic detergent. Salt precipitation removes proteins and other impurities. Absolute ethanol (EtOH) or isopropanol (IP) are typically employed to precipitate gDNA from its aqueous solution when sodium ions are present. The volume of EtOH or IP (1–2× the volume of supernatant containing gDNA), the incubation temperature (−20°C), and the duration (10–60 min) utilized for gDNA precipitation all vary greatly. IP and EtOH can also be used to wash and purify gDNA [32]. RNase H enzyme is frequently employed to remove ribonucleic acid (RNA), while CTAB is virtually always included in lysis solutions [33]. In this study, we evaluated the fungal gDNA yield, integrity, and amplification success of different gene sequences using the two different commercial kit methods in comparison to our modified CTAB method.

## 2 Materials and methods

### 2.1 Isolation of fungal species from dry fruits

Dry fruits (walnuts, apricots, almonds, and cashew nuts) were purchased from the local markets, and 100 g were disinfected separately by immersion in 0.1% sodium hypochlorite solution for 1 min (60 s). Seven pieces of dry fruits (unshelled) were plated on Potato Dextrose Agar plates and then incubated for 7 days at 27°C. Liquid cultures of fungi were grown in potato dextrose broth (PDB) in a shaking incubator at 27°C with continuous shaking (160 revolutions per minute [RPM]) for 7 days [34]. The fungal species were identified through a combination of morphological assessment and molecular techniques, with the identification process guided by the comprehensive reference “Fungi and Food Spoilage” by Pitt and Hocking [35], which is a seminal work in the field of mycological studies related to food spoilage.

### 2.2 Harvesting mycelium and spore suspension

Fungal strains were inoculated into 50 mL of PDB in Erlenmeyer flasks (100 mL) and incubated for 3 days at 27°C and after incubation, mycelium was filtered through a Buchner funnel, washed thoroughly twice with distilled water, and again filter dried. The above procedure was used to prepare the mycelium, which was then weighed in Eppendorf tubes before DNA extraction. The weight of the mycelium utilized to extract the DNA was 20 mg. Fungal isolates of spore suspensions were made from 7-day-old cultures in PDB at 27°C using phosphate buffered saline, pH 7.4, and 0.05% Tween 80. The upper homogeneous suspensions were then transferred to sterile tubes and adjusted to a concentration of approximately 10^6^ conidia/mL by hemocytometer counting after heavy particles were allowed to settle for 10–15 min [36].

### 2.3 DNA extraction protocol (modified CTAB)

#### 2.3.1 Step 1: Preparation of extraction Buffer A (Table 1)

**Table 1:** Stock and working solution of CTAB DNA extraction buffer (Buffer A)

Chemical	Stock solution	Working solution	Mass or volume
CTAB	2%	2%	5 g
NaCl	5 M	1.5 M	75 mL
EDTA (pH = 8)	0.5 M	20 mM	10 mL
Tris-HCL	1 M	200 mM	20 mL
Water			Raised to 250 mL

- Mix 25 mL of 1 M Tris (pH 8.0), 75 mL of 5M NaCl, 10 mL of 0.5M ethylenediaminetetraacetic acid (EDTA), and 5 g of CTAB.
- Bring the final volume to 250 mL with milli-Q water.
- Preheat Buffer A in a water bath at 55°C for 15–20 min until the solution becomes translucent.

#### 2.3.2 Step 2: Preparation of extraction Buffer B (Table 1)

- Prepare a solution of 1% (w/v) polyvinylpyrrolidone (PVP).
- Filter the solution for sterilization and removal of particulates.
- Autoclave the solution and store it at −20°C until further use.

#### 2.3.3 Step 3: Mycelium or spore suspension grinding (cell crushing methods)

- Weigh 20 mg of mycelium tissues or spore suspension (10^6^ conidia/mL).

##### 2.3.3.1 Option 1: Mortar and pestle

- Grind the mycelium or spore suspension with 900 µL of Buffer A and 100 µL of Buffer B until a paste is formed.
- Transfer the paste to 2 mL Eppendorf tubes for immediate processing.

##### 2.3.3.2 Option 2: Liquid nitrogen

- Chill the mortar and pestle at −20°C.
- Grind the mycelium or spore suspension to a powdered form with liquid nitrogen.
- Transfer the powder to 2 mL Eppendorf tubes for immediate processing.

##### 2.3.3.3 Option 3: Micro pestle grinding

- Transfer 20 mg of mycelium or spore suspension to 5 mL tubes.
- Add 900 µL of Buffer A and 100 µL of Buffer B.
- Pulverize the mixture using an Eppendorf micro pestle and proceed with DNA extraction.

#### 2.3.4 Step 4: Cell lysis and DNA extraction

- Add 400 µL of Buffer A and 60 µL of Buffer B to the ground mycelium tissues or spore suspension.
- Incubate at 57°C for 35 min in a water bath, inverting the tubes every 10 min.
- Centrifuge the mixture at 10,000 rpm for 10 min at 4°C to remove cell debris.

#### 2.3.5 Step 5: DNA purification

- Transfer the supernatant to a fresh tube.
- Add an equal volume of chloroform: isoamyl alcohol (24:1 v/v) and mix gently by inversion for 15 min at 25°C.
- Centrifuge the mixture at 10,000 rpm for 12 min at 4°C.
- Carefully collect the aqueous phase.

#### 2.3.6 Step 6: DNA precipitation

- Add 1.35 volumes of chilled IP (stored at −20°C) to the aqueous phase.
- Mix by inverting the tube and incubate at −20°C for 30 min.
- Centrifuge at 10,000 rpm for 5 min at 4°C.

#### 2.3.7 Step 7: DNA washing

- Wash the DNA pellet with 70% EtOH.
- Centrifuge at 10,000 rpm for 5 min at 4°C.
- Remove EtOH entirely by centrifugation and air-dry the DNA pellet for 10 min.

#### 2.3.8 Step 8: DNA resuspension

- Dissolve the DNA pellet in 50 µL of Tris-EDTA buffer.
- Vortex briefly to resuspend the DNA.

#### 2.3.9 Step 9: Optional DNA cleanup (if needed)

- Use a commercial purification kit if additional purification is required.
- Follow the manufacturer’s protocol for the cleanup procedure.

#### 2.3.10 Step 10: DNA quantification and visualization

##### 2.3.10.1 Qubit measurement (optional but recommended)

- Quantify the DNA using a Qubit fluorometer to assess the concentration and purity of the extracted DNA.
- Measure the Qubit/Nanodrop yield ratio to ensure DNA purity.

##### 2.3.10.2 Nanodrop measurement

- Quantify the DNA using a NanoDrop spectrophotometer to measure absorbance at 260/280 nm and 260/230 nm.

##### 2.3.10.3 Gel electrophoresis

- Perform gel electrophoresis using a 0.8% agarose gel stained with EtBr.
- Visualize the DNA bands using a gel documentation system.

### 2.4 Commercial isolation kit methods

Fungal gDNA was extracted by using two commercially available kit-based methods. The Firs kit-based method was HiPura^TM^ Fungal DNA Purification Kit (HiMedia Laboratories Pvt. Ltd), which is designated for isolating DNA from fungal species with spin column procedures. The second kit-based method was DNeasy Plant Mini Kit (QIAGEN) designated for isolating DNA from plant leaves. While extracting fungal gDNA using the commercial kits cited above, the manufacture’s protocols were strictly followed (accessed on the website of Himedia and Qiagen).

### 2.5 Quantification and visualization of DNA

To obtain a precise estimation of the amount of gDNA extracted, we used a NanoDrop spectrophotometer (Thermo Scientific, Wilmington, DE, USA) to measure the important parameters of gDNA quality, including the ratio of absorbance at 260/280 nm and 260/230 nm, essential indicators of gDNA quality. For gDNA band analysis, we used an agarose gel (0.8%) stained it with 0.5 µg/mL EtBr. About 5 µL of isolated gDNA was loaded in the wells after addition of 1 µL 5× DNA loading dye (HiMedia Laboratories). The gel was then electrophoresed for an hour at 80 V in 200 mL of 1× tris-acetate-EDTA buffer (HiMedia Laboratories). Using a gel documentation system (GenoSens 2000) with nucleic acid visualization (embedded touch screen PC), gel images were obtained. Each method was repeated in triplicate for precise results.

### 2.6 PCR amplification of multilocus gene sequences

gDNA of fungal isolates from dry fruits were used as template for multilocus gene amplification, and different gene sequences were selected and amplified with a set of primers (Table 2). The reaction mixture of 50 µL contained 50 ng of template gDNA, 1× PCR buffer, 1.5 mM of MgCl_2_, 200 μM of deoxynucleoside triphosphates, 0.20 μM of each primer, and 2 U of Taq polymerase. Controls were run with every series of amplifications to test for the presence of contaminants. The PCR products were separated on a 1.5 agarose gel stained with EtBr. The electrophoresis was maintained at 80 V for 30 min. By comparing the size of the gene fragments to a DNA marker of 100–1,500 base pairs (bp), the size of the amplified gene sequences was determined.

**Table 2:** List of genes, primers, and primer sequences used for amplification of nuclear housekeeping genes and protein coding genes

Gene (Primer)	Forward	Reverse	Reference
Internal transcribed spacer (ITS; ITS1, ITS4)	5′-TCCGTAGGTGAACCTGCGG-3′	5′-TCCTCCGCTTATTGATATG-3′	[65]
Translation elongation factor 1-alpha (tef1-α; EF595F, EF1160R)	5′-CGTGACTTCATCAAGAACATG-3′	5′-CCGATCTTGTAGACGTCCTGC-3′	[66]
RNA polymerase II second largest subunit (RPB2; bRPB2-6.9R, bRPB2-11R1)	5′-TGGACNCAYTGYGARATYCAYCC-3′	5′-TGGATYTTGTCRTCCACCAT-3′	[67]
28S ribosomal DNA (rDNA) (NS1, NS2)	5′-ACCCGCTGAACTTAAGC-3′	5′-CGCCAGTTCTGCTTACC-3′	[68]
18S ribosomal DNA (rDNA) (NS1, NS2)	5′-GTAGTCATATGCTTGTCTC-3′	5′-5GGCTGCTGGCACCAGACTTGC-3′	[65]
Calmodulin	5′-ACATTTGCATCCCCAGC-3′	5′-TGCACTTCCCGACATCATCC-3′	[69]

### 2.7 Statistical analysis

Statistical analysis was conducted to rigorously assess the results obtained from the gDNA extraction experiments. Data curation was meticulously carried out to ensure the accuracy and reliability of the dataset. Analysis of variance (ANOVA), a widely used statistical method to compare means across multiple groups, was employed to determine the significance of any differences observed among the experimental groups. The statistical analysis was performed using Stat SP, a specialized statistical analysis software known for its robustness and versatility in handling complex datasets. This software was chosen for its ability to effectively execute ANOVA and other required statistical tests required for this study.

Following the ANOVA, *post hoc* tests were conducted to further scrutinize any significant differences identified among the experimental groups. *Post hoc* ANOVA tests are essential for pinpointing specific group differences and elucidating the nuances within the data that may not be apparent from the initial ANOVA alone.

## 3 Results

### 3.1 Efficiency of different DNA extraction methods

**Figure 1:**
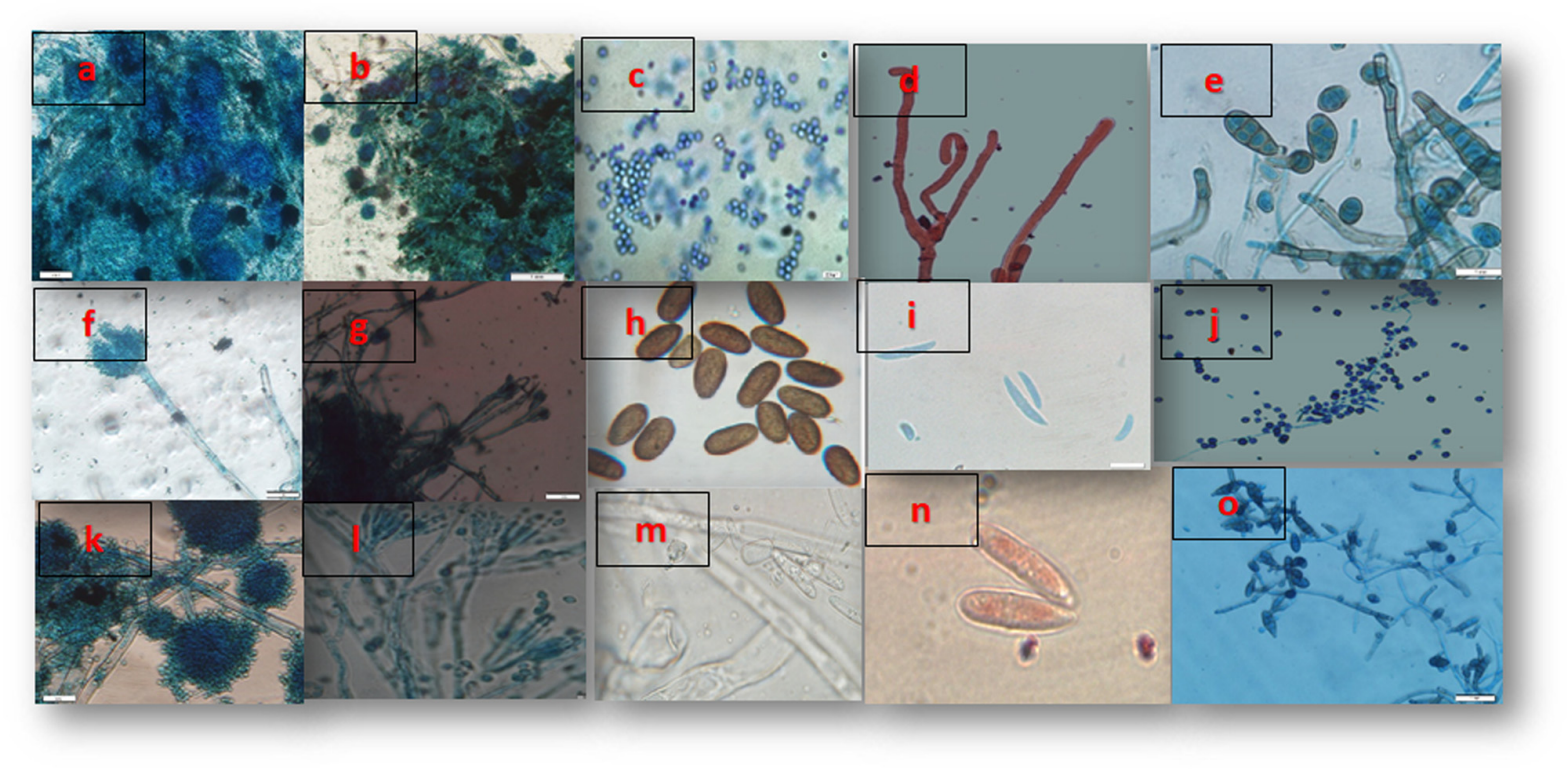
(a) Conidial heads of *Aspergillus*; (b) Conidia and conidiophores of *Penicillium*; (c) Conidiophores with conidia, likely *Aspergillus*; (d) Septate hyphae of *Fusarium*; (e) Conidia and conidiophores, *Alternaria*; (f) Conidial head of *Aspergillus*; (g) Mycelium and spores, *Penicillium*; (h) Oval conidia of *Alternaria*; (i) Sickle-shaped conidia of *Fusarium*; (j) Conidia in chains, *Penicillium*; (k) Conidial heads of *Aspergillus*; (l) Hyphae with conidia, *Penicillium*; (m) Hyphae of *Fusarium*; (n) Macroconidia of *Fusarium*; (o) Hyphae with conidia of *Alternaria*.

We investigated the efficiency of a modified CTAB method for DNA extraction from fungal species, including *Aspergillus*, *Penicillium*, *Alternaria*, *Dothiorella*, and *Fusarium* (Figure 1). The isolates were obtained from dry fruits post 0.1% sodium hypochlorite treatment. After morphological identification, a standardized lysis method was employed. Linear regression analysis demonstrated a strong correlation coefficient of determination (*R* ^2^ = 0.9870) between mycelium weight (mg) and DNA concentration nanograms per microliter (ng/µL) across diverse fungal isolates, signifying the efficacy of the modified CTAB method (*p* < 0.05). However, a decrease in gDNA yield was noted when spore suspension was used for gDNA extraction.

### 3.2 Comparison of DNA extraction methods

**Figure 2:**
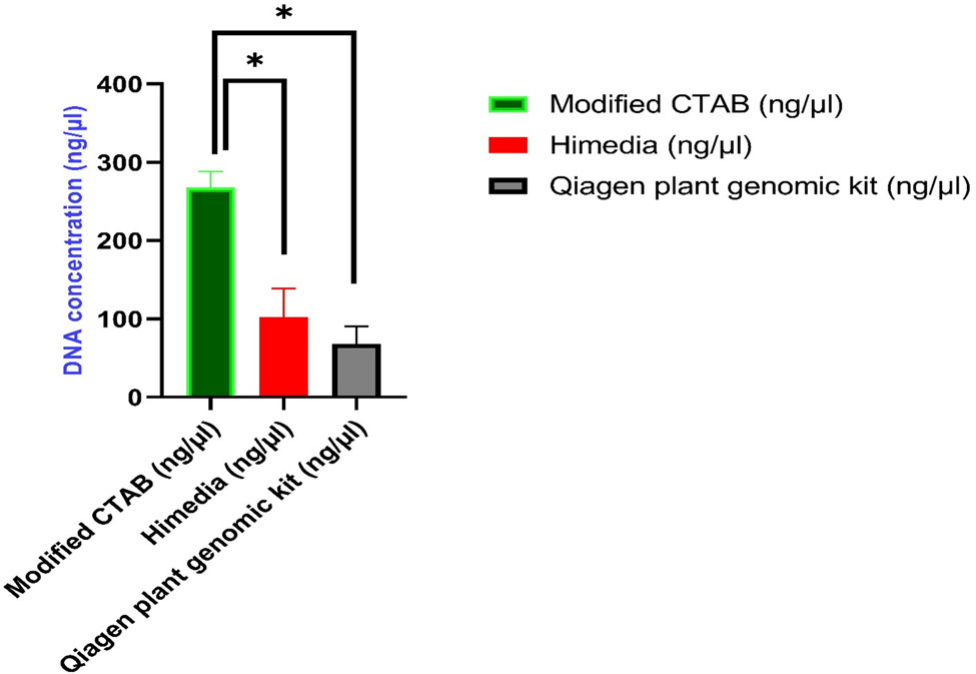
Different fungal isolates (*n* = 40) were checked for DNA concentration (ng/µL) from mycelium and from spore suspension using three extraction methods. Modified CTAB presented a significant yield (mean value ± SEM, 267.67 ± 20.69) as compared to other kit protocols (*marker means, *p* < 0.001) analyzed by using one way-ANOVA and Dunnett’s multiple comparisons test.

In the present study, modified CTAB gDNA extraction method exhibited a substantial mean difference in DNA yield compared to alternative methods, namely, Himedia and Qiagen plant genomic kit (Figure 2). The statistical analysis, employing Dunnett’s multiple comparisons test, revealed a significant increase in DNA yield with our modified CTAB protocol when compared to Himedia (mean difference: 165.7, 95% confidence interval, CI: 78.86–252.5) and Qiagen plant genomic kit (mean difference: 199.6, 95% CI: 112.7–286.4). The adjusted *p*-values (<0.001) underscore the robustness of these differences. These findings emphasize the efficacy of our modified CTAB method in extracting DNA, highlighting its potential superiority over the tested alternatives (Table 1, Figure 2).

### 3.3 Effectiveness of different cell crushing method

**Figure 3:**
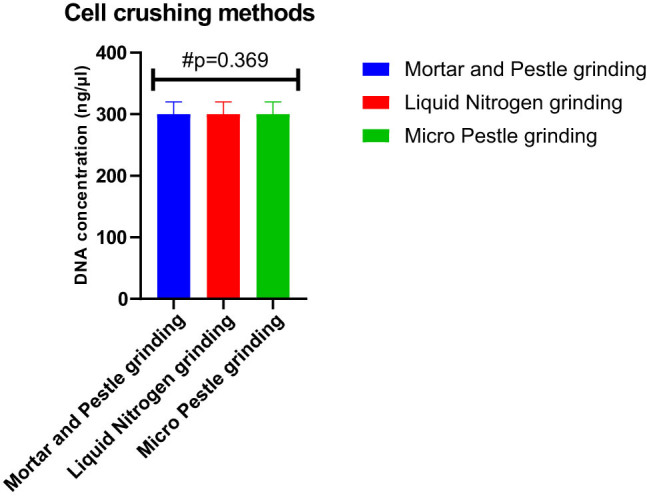
Tukey’s multiple comparisons test for cell crushing methods, no significant differences were observed between the methods, as indicated by non-significant *p*-values exceeding the conventional significance threshold of 0.05.

Absorbance ratios for various cell crushing methods ranged from 1.85 to 1.93 for all the methods employed, indicating no significant impact on absorbance ratios. This study employs Tukey’s multiple comparisons test to assess the differences in DNA yield among three distinct cell crushing methods: mortar pestle, liquid nitrogen, and micropestle grinding. The analysis focuses on the mean differences in DNA concentration measured in nanograms per microliter (ng/µL) along with 95% CI and adjusted *p*-values. Despite the observed mean differences, the statistical comparisons did not reveal significant variations between the mortar pestle method vs liquid nitrogen, mortar pestle method vs micro pestle grinding, and liquid nitrogen vs micro pestle grinding. The adjusted *p*-values for these comparisons were found to be 0.919, 0.926, and 0.726, respectively (Figure 3).

### 3.4 Analysis of DNA size and DNA concentration

The DNA fragment sizes observed across the fungal isolates range from 30 to 60 kilobase pairs (kb), reflecting varying degrees of DNA integrity. Larger DNA fragments, particularly those observed in isolates such as *Aspergillus welwitschiae* and *Dothiorella gregaria* (60 kb), suggest the presence of high molecular weight DNA, which is more conducive to downstream applications such as sequencing. Conversely, smaller DNA sizes, like the 30 kb observed in *Aspergillus tabacinus* (AZ9), may indicate some degree of DNA fragmentation, although they still represent a considerable amount of intact genetic material.

DNA concentrations across the isolates range from approximately 210–298 ng/µL. There appears to be a positive correlation between DNA size and concentration, with higher concentrations typically associated with larger DNA fragments. For instance, *Aspergillus niger* (N72) shows a DNA size of 55 kb coupled with a concentration of 256.36 ng/µL. This correlation implies that isolates with less fragmented, higher quality DNA tend to yield greater quantities, which is advantageous for molecular studies requiring substantial and intact DNA.

### 3.5 PCR amplification of multilocus genes (MLST)

The template gDNA used for PCR amplification of these gene sequences was extracted by our modified CTAB method (Table 3). All five gene sequences, with amplicon sizes ranging from 422 to 929 bp, were effectively amplified from various fungus species obtained from dried fruits.

**Table 3:** Fungal isolates with identified species, amplified gene loci, DNA sizes, and DNA concentrations

Isolate	Species identified	Amplified gene loci					DNA size (kb)	DNA concentration (ng/µL)
		ITS	tef1-α	RPB2	28S rDNA	18S rDNA		
10WHITE	*Fusarium acuminatum*	**Yes**	**Yes**	**Yes**	**Yes**	**Yes**	35	**210.12**
AG10	*Penicillium nordicum*	**Yes**	**Yes**	**Yes**	**Yes**	**Yes**	45	**230.23**
AG9	*Penicillium viridicatum*	**Yes**	**Yes**	**Yes**	**Yes**	**Yes**	55	**250.98**
AP4	*Penicillium neoechinulatum*	**Yes**	**Yes**	**Yes**	**Yes**	**Yes**	40	**212.36**
AR2	*Aspergillus niger*	**Yes**	**Yes**	**Yes**	**Yes**	**Yes**	50	**219.26**
AV1	*Aspergillus welwitschie*	**Yes**	**Yes**	**Yes**	**Yes**	**Yes**	60	**231.32**
AV9	*Aspergillus versicolor*	**Yes**	**Yes**	**Yes**	**Yes**	**Yes**	55	**236.98**
AV91	*Aspergillus versicolor*	**Yes**	**Yes**	**Yes**	**Yes**	**Yes**	55	**298.36**
AW0	*Penicillium viridicatum*	**Yes**	**Yes**	**Yes**	**Yes**	**Yes**	45	**256.32**
AW1	*Penicillium nordicum*	**Yes**	**Yes**	**Yes**	**Yes**	**Yes**	35	**265.85**
AW4	*Penicillium polonicum*	**Yes**	**Yes**	**Yes**	**Yes**	**Yes**	40	**272.31**
AW5	*Penicillium albocoremium*	**Yes**	**Yes**	**Yes**	**Yes**	**Yes**	50	**212.39**
AYWG1	*Penicillium solitum*	**Yes**	**Yes**	**Yes**	**Yes**	**Yes**	55	**213.96**
AZ12	*Aspergillus austroafricanus*	**Yes**	**Yes**	**Yes**	**Yes**	**Yes**	45	**236.36**
AZ13	*Aspergillus versicolor*	**Yes**	**Yes**	**Yes**	**Yes**	**Yes**	50	**251.32**
AZ14	*Aspergillus tubingensis*	**Yes**	**Yes**	**Yes**	**Yes**	**Yes**	60	**214.01**
AZ9	*Aspergillus tabacinus*	**Yes**	**No**	**Yes**	**Yes**	**No**	30	**226.13**
F10R10	*Fusarium lateritium*	**Yes**	**Yes**	**Yes**	**Yes**	**Yes**	40	**225.19**
F21R222	*Penicillium citrinum*	**Yes**	**Yes**	**Yes**	**Yes**	**Yes**	55	**211.12**
F21R223	*Penicillium chrysogenum*	**Yes**	**Yes**	**Yes**	**Yes**	**Yes**	45	**220.23**
F23R23	*Aspergillus protuberus*	**Yes**	**Yes**	**Yes**	**Yes**	**Yes**	60	**240.98**
F23R24	*Penicillium commune*	**Yes**	**Yes**	**Yes**	**Yes**	**Yes**	50	**222.36**
F3R3	*Aspergillus flavus*	**Yes**	**Yes**	**Yes**	**Yes**	**Yes**	55	**219.23**
FA2Y	*Penicillium thymicola*	**Yes**	**Yes**	**Yes**	**Yes**	**Yes**	35	**231.39**
FA6G	*Aspergillus amoenus*	**Yes**	**Yes**	**Yes**	**No**	**No**	45	**236.91**
N71	*Geotrichum candidum*	**Yes**	**Yes**	**Yes**	**Yes**	**Yes**	55	**298.33**
N72	*Aspergillus niger*	**Yes**	**Yes**	**Yes**	**Yes**	**Yes**	55	**256.36**
N724	*Aspergillus niger*	**Yes**	**Yes**	**Yes**	**Yes**	**Yes**	55	**265.82**
N726	*Aspergillus niger*	**Yes**	**Yes**	**Yes**	**Yes**	**Yes**	50	**282.31**
N73	*Aspergillus brasiliensis*	**Yes**	**Yes**	**Yes**	**Yes**	**No**	60	**252.39**
N74	*Aspergillus welwitschiae*	**Yes**	**Yes**	**Yes**	**Yes**	**Yes**	45	**253.96**
NY01	*Penicillium cordubense*	**Yes**	**Yes**	**Yes**	**No**	**Yes**	45	**286.36**
NY15	*Penicillium hordei*	**Yes**	**Yes**	**Yes**	**Yes**	**Yes**	50	**261.32**
NY3	*Penicillium lapidosum*	**Yes**	**Yes**	**Yes**	**Yes**	**Yes**	50	**274.01**
NY5	*Penicillium verrucosum*	**Yes**	**Yes**	**Yes**	**Yes**	**Yes**	50	**226.13**
P2	*Penicillium thomii*	**Yes**	**Yes**	**Yes**	**Yes**	**Yes**	50	**225.19**
R4F4	*Penicillium crustosum*	**Yes**	**Yes**	**Yes**	**Yes**	**Yes**	45	**246.12**
R5F5	*Geotrichum candidum*	**Yes**	**Yes**	**Yes**	**Yes**	**Yes**	45	**236.21**
R6F6	*Geotrichum candidum*	**Yes**	**Yes**	**Yes**	**Yes**	**Yes**	60	**256.81**
NY6	*Dothiorella gregaria*	**Yes**	**Yes**	**Yes**	**Yes**	**Yes**	50	**297.36**
*P132*	*Fusarium oxysporum*	**Yes**	**Yes**	**Yes**	**Yes**	**Yes**	60	**286.83**
*NY65*	*Dothiorella gregaria*	**Yes**	**Yes**	**Yes**	**Yes**	**Yes**	35	**298.32**
*FAYRF*	*Aspergillus fumigatus*	**Yes**	**Yes**	**Yes**	**Yes**	**Yes**	45	**293.45**

Note: “Yes” indicates successful amplification of the respective gene sequence, while “No” indicates that the gene sequence was not amplified. DNA sizes are provided in kilobases (kb) and reflect the integrity of the isolated DNA.

Later these genes were sequenced using both forward and reverse primers (Table 2) and chromatographs were analyzed by FinchTV. All gene sequences had showed high-quality chromatographs. Query sequences were submitted for BLASTn search in national center for biotechnology information for sequence identity and species identification. Different fungal isolates of our study showed sequence similarity of 95–100% with different *Aspergillus* species: 98–100% sequence similarity with *Penicillium* species, 93–100% sequence similarity with *Fusarium* species, 99% sequence similarity with *Mucor* species, 100% sequence similarity with *Galactomyces* species, 99.8% sequence similarity with *Dothiorella* species, and 99.45% sequence similarity with *Alternaria* species using five housekeeping gene markers (Table 2) for molecular identification systematic analysis. Remarkably, the majority of fungal isolates exhibited successful amplification across all targeted gene loci, including ITS, tef1-α, RPB2, 28S rDNA, and 18S rDNA. This successful amplification is indicative of the robustness of the modified CTAB DNA extraction and amplification protocols employed in this study. Particularly, isolate 10WHITE (*Fusarium acuminatum*) displayed successful amplification for all gene loci, accompanied by a substantial DNA concentration of 210.12 ng/µL. Similarly, various *Aspergillus* and *Penicillium* species consistently exhibited positive results across the amplified gene loci, showcasing the reliability and effectiveness of the extraction methodology. Significantly, the isolate AV91 (*Aspergillus versicolor*) demonstrated a high DNA concentration of 298.36 ng/µL, further emphasizing the efficiency of the DNA extraction protocol. However, isolate AZ9 (*Aspergillus tabacinus*) exhibited a lack of amplification for the tef1-α and 18S rDNA gene loci, suggesting potential variability in the DNA extraction efficiency for this specific isolate. Overall, the consistently successful amplification across various fungal isolates, coupled with notable DNA concentrations, attests to the effectiveness of the modified CTAB protocol employed in this study for extracting high-quality fungal DNA suitable for downstream molecular applications (Table 3).

## 4 Discussion

This study provides a detailed evaluation of the modified CTAB protocol for fungal gDNA extraction, highlighting its potential as a superior alternative to commercial kits, such as those offered by HiMedia and Qiagen, particularly for advanced molecular applications including MLST and next-generation sequencing (NGS). The extraction of high-quality DNA from fungi poses significant challenges due to the chitin-rich composition of their cell walls, which are highly resistant to degradation and complicate the crucial initial step of cell lysis (Figure 3). Chitin, a durable and fibrous biopolymer, imparts considerable structural rigidity, necessitating the development of enhanced extraction methods to effectively recover intact gDNA [37,38,39]. The difficulty in disrupting these cell walls frequently results in DNA fragmentation or degradation, which can severely impact the integrity and purity of the extracted DNA, both of which are essential for the accuracy of downstream applications such as PCR and sequencing [40].

In response to these challenges, there is a growing need for extraction protocols that can effectively overcome the barriers posed by the chitinous cell walls of fungi, ensuring the recovery of high-quality, intact DNA (Table 3). The modified CTAB protocol has been specifically developed to address the limitations commonly associated with commercial DNA extraction kits, particularly issues related to contamination and the presence of PCR inhibitors, which can critically compromise the quality of the extracted DNA [41]. The inclusion of 1% PVP in the modified CTAB protocol (Table 1) significantly enhances the binding and removal of inhibitory compounds, thereby preventing their interference during the DNA extraction process [31,42,43]. This modification is particularly crucial for ensuring the purity of the extracted DNA, as demonstrated in studies focusing on applications such as NGS and MLST, where even minimal impurities can lead to significant errors in sequencing data and phylogenetic analyses [44].

Although the sodium dodecyl sulfate-based method offers benefits in terms of simplicity and cost, the CTAB method is chosen when the research demands the highest possible DNA purity, yield, and integrity, particularly for complex or challenging samples. This makes CTAB the method of choice for studies where these factors are critical for the success of downstream molecular analyses [45].

Recent studies have further validated the effectiveness of the modified CTAB protocol across various contexts. For instance, the use of modified CTAB protocols has proven effective in extracting high-molecular-weight DNA from ferns, a group known for their large and complex genomes. These protocols, which minimize mechanical disruption during lysis to prevent DNA shearing, have successfully yielded high-purity DNA, essential for long-read sequencing technologies [46]. Similarly, modified CTAB protocols have been adapted for high-quality RNA extraction from challenging samples such as algae and plants, where the presence of secondary metabolites complicates the process [47,48]. These adaptations underscore the versatility of the modified CTAB protocol in handling various types of complex biological materials, further supporting its application in fungal DNA extraction.

The proven efficacy of the modified CTAB protocol in producing high-purity, contaminant-free DNA underscores its suitability for applications where DNA integrity is paramount. This positions the modified CTAB protocol as particularly advantageous for researchers and clinicians who require stringent and reliable DNA extraction methods for complex fungal samples, especially in contexts demanding high precision, such as in NGS and MLST [49,50].

Furthermore, this study contributes to the expanding body of research emphasizing the critical role of fungal life stages in the efficacy of DNA extraction protocols. Consistent with previous findings, our research demonstrated that spore suspensions yielded significantly lower DNA concentrations compared to mycelium, aligning with studies that have highlighted the structural complexities of spores, including their thicker and more resilient cell walls, which necessitate optimized extraction protocols to achieve maximum DNA yield [51,52].

Moreover, the robust performance of the modified CTAB protocol across diverse fungal species and life stages highlights the importance of customized strategies in obtaining high-quality DNA, which is essential for accurate molecular analyses and subsequent applications in research and diagnostics [53]. The clinical implications of these findings are particularly noteworthy in the context of fungal pathogen detection, where the accuracy and timeliness of diagnostics are critical [54]. Rhinosinusal aspergilloma, caused by Aspergillus species, remains a diagnostic challenge due to the limitations inherent in traditional methods [55]. The high-purity DNA extracted using the modified CTAB protocol provides a robust foundation for developing more precise and sensitive fungal pathogen detection methods, particularly in the diagnosis of rhinosinusal aspergilloma, where accurate DNA extraction is crucial for effective clinical outcomes [56].

Furthermore, the modified CTAB protocol’s application extends beyond the detection of aspergilloma, offering broader utility in the diagnosis and management of a range of invasive fungal infections. These infections are increasingly prevalent in immunocompromised populations, highlighting the urgent need for effective diagnostic strategies [57]. The ability to reliably extract high-quality DNA from diverse fungal pathogens positions the modified CTAB protocol as a valuable tool in the evolving landscape of clinical diagnostics and fungal disease management [58].

In addition to its application in molecular diagnostics, the modified CTAB protocol has been employed in genotyping-by-sequencing to map meiotic crossovers in plants, further illustrating its versatility and reliability across different biological systems [59]. The adaptability of the modified CTAB protocol to various contexts, along with its consistent ability to yield high-quality DNA, underscores its importance as a fundamental tool for researchers working with complex biological samples.

To optimize DNA extraction from samples with high starch, polysaccharide, and polyphenolic content, strategic modifications to the CTAB protocol have demonstrated significant efficacy. For samples with elevated starch content, increasing the NaCl concentration in the extraction buffer to 2.5 M markedly enhances both the yield and purity of the extracted DNA. This adjustment is particularly effective in dissociating polysaccharides from nucleic acids, thereby preventing the co-precipitation of starch with DNA and improving the overall quality of the extraction.

In dealing with polysaccharide-rich samples, several methodological enhancements have been proposed. One effective strategy involves the preparation of crude chromatin prior to DNA extraction, using a CTAB/high-salt buffer combined with 3% (w/v) sarcosyl, which facilitates the removal of polysaccharides that are prone to co-precipitating with DNA and may interfere with downstream applications such as PCR. Alternatively, treating the extract with water-saturated ether in the presence of 1.25 M NaCl, or purifying the DNA by adsorption onto a silica suspension in guanidine thiocyanate, has proven effective in eliminating these contaminants.

For samples high in polyphenolic compounds, the incorporation of 1–2% (w/v) PVP with molecular weights of 10,000 or 40,000 into the extraction buffer effectively neutralizes phenolic compounds, which otherwise pose a risk of inhibiting enzymatic reactions in subsequent molecular analyses. Additionally, in cases involving highly viscous mucilage, a high concentration of sarcosyl in the extraction buffer can be employed to precipitate polysaccharides, thereby reducing viscosity and enhancing the DNA yield.

Furthermore, to mitigate the oxidation of polyphenols, particularly in phenol-rich samples, the inclusion of antioxidants such as diethyldithiocarbamic acid (4 mM) and ascorbic acid (5 mM), along with 2% (w/v) PVP40, has been found beneficial. These modifications collectively preserve the integrity of the extracted DNA, ensuring its suitability for subsequent molecular applications [60,61,62,63,64].

## 5 Conclusion

Based on anticipated gDNA yield and quality, extraction time, cost effectiveness, successful amplification, and waste management, our findings serve as a guide for selecting techniques of gDNA extraction from fungal species associated with dried fruits. This research report describes a modified CTAB DNA extraction protocol for fungi that is cost effective and rapid. The protocol uses mortar and pestle with Buffer A and Buffer B to crush the cells, eliminating the need for liquid nitrogen grinding and bead beating. The modified CTAB protocol also allows for the sterilization of chemicals and lysis buffer, removing any unwanted bacterial or fungal contamination that might interfere with PCR amplification. This protocol is flexible enough to give a white colored gDNA pellet, which is beneficial for downstream PCR amplification and MLST analysis. The protocol was tested using PCR primers from earlier studies and showed enhanced rate of gene amplification for a variety of fungal species, indicating its potential use for nucleic-based fungal disease diagnosis such as fish fungal diseases, plant pathogens, fruit rot associated pathogens, and human fungal diseases.
